# Fourth Surviving Sepsis Campaign’s hemodynamic recommendations: a step forward or a return to chaos?

**DOI:** 10.1186/s13054-017-1708-z

**Published:** 2017-05-30

**Authors:** Glenn Hernández, Jean-Louis Teboul

**Affiliations:** 1 Departamento de Medicina Intensiva, Facultad de Medicina, Pontificia Universidad Católica de Chile, Santiago, Chile; 2 Service de Réanimation médicale, Hôpitaux universitaires Paris-Sud, Assistance Publique-Hôpitaux de Paris, Hôpital de Bicêtre, 78 rue du Général Leclerc, Le Kremlin-Bicêtre, F-94270 France

Since the first publication of the Surviving Sepsis Campaign (SSC) guidelines [1], a corpus of contradictory evidence as well as physiological objections on critical aspects of the hemodynamic resuscitation have emerged over time [2–4]. The most recent SSC guidelines [5] have radically moved from a structured bundle approach to extremely broad recommendations, leaving fundamental pieces of care without clear-cut guidance for attending physicians, especially for monitoring and treatment. We wish to critically analyze potential negative consequences of some SSC’s hemodynamic recommendations and to discuss neglected areas.

## Fluids

It is now recommended to infuse at least 30 mL/kg of IV crystalloids within the first 3 h of resuscitation of sepsis-induced hypoperfusion. We are concerned by both the predefined volume and timeframe. First, all septic patients do not exhibit the same degree of hypovolemia. For instance, abdominal sepsis inducing massive internal or external fluid losses is generally not equivalent to community-acquired pneumonia in terms of volume deficit. Deliberate administration of 30 mL/kg of fluids in patients with pneumonia with cardiovascular comorbidities might eventually result in pulmonary edema and hasten the need for mechanical ventilation. Initial fluid resuscitation should be individualized according to several elements, including clinical signs of hypovolemia, body temperature, pulse pressure, age, comorbidities, and sepsis origin. If hypovolemia is assumed to be a major component of hypoperfusion, fluids should be infused as a rapid fluid bolus to increase mean systemic filling pressure, venous return, and stroke volume [6]. The same amount of fluids infused in 3 h cannot have a measurable effect on systemic blood flow in this context. Second, early reassessment of hemodynamics is a fundamental aspect of management of patients with shock [7]. It is unreasonable to wait for 3 h—as it is suggested in the SSC guidelines [5]—before reassessing the effects of the initial fluid therapy.

## Vasopressors

From the last version of the SSC publication [5], it is unclear when norepinephrine should be initiated. The reader could understand that the decision should be made only at the time of the first reassessment (3 h). One major characteristic of septic shock is vasoplegia, where the need of a vasopressor is mandatory since fluid resuscitation alone cannot restore vascular tone and thus cannot completely correct profound hypotension [8], which is an event associated with mortality [9]. In addition, sepsis-induced vasoplegia results in a dramatic fall in diastolic arterial pressure (DAP), which represents the upstream pressure for perfusion of the left ventricle. A low DAP, especially in the context of tachycardia, can be an easy bedside tool to identify patients who need early initiation of a vasopressor. This was mentioned in the previous publication of the SCC [10] but disappeared inexplicably in the most recent one [5]. Early initiation of a vasopressor not only can rapidly correct hypotension in case of low vascular tone but also can avoid harmful fluid overload.

## Perfusion monitoring

After several recent negative trials testing the use of central venous oxygen saturation (ScvO_2_) as a target for early resuscitation of septic shock [4], the SSC has abandoned its initial recommendation to include ScvO_2_ as part of standard monitoring. This could lead to a loss of confidence in the value of multimodal perfusion monitoring to assess the adequacy of systemic blood flow in relation to oxygen demand. This is profoundly regrettable since a low ScvO_2_ generally reveals an inadequate cardiac output and suggests the presence of a hypoperfusion context in case of a persistent hyperlactatemia (“flow-sensitive” hyperlactatemia) [11].

## Lactate normalization as a resuscitation target

Normalization of lactate is recommended by the SSC as a resuscitation goal since it is assumed that tissue hypoxia is the main source of lactate production. However, there are several unresolved concerns about the role of lactate as an appropriate resuscitation target. First, besides hypoperfusion, adrenergic-driven aerobic lactate production and impaired hepatic lactate clearance have been suggested as important contributors to persistent hyperlactatemia [12]. Since non-hypoperfusion-related causes of hyperlactatemia might predominate in an unknown number of patients, aiming at strictly normalizing lactate might lead to excessive resuscitation with inherent fluid and vasopressor overload, and eventually to increased morbidity and mortality. Second, the dynamics of recovery of blood lactate exhibits a complex pattern and might not, therefore, be the best tool for real-time assessment of the effect of hemodynamic resuscitation [12–14]. A recent study by Hernandez et al. [14] demonstrated that only 50% of septic shock survivors normalize lactate during the first 24 h of management. Variables such as ScvO_2_, central venous-arterial PCO_2_ gradient (P(cv-a)CO_2_), and peripheral (skin) perfusion markers exhibit a very fast normalization rate in relation to systemic flow optimization, whereas blood lactate shows a biphasic response with an initial rapid improvement in parallel with the above-mentioned variables, followed by a much slower trend thereafter [14]. Thus, a concomitant low ScvO_2_, or high P(cv-a)CO_2_, or abnormal peripheral perfusion defines a “hypoperfusion context” in which increasing systemic blood flow may contribute to blood lactate decrease. Applying these criteria to septic hyperlactatemic patients in a recent proof-of-concept study clearly differentiated two subpopulations of patients exhibiting markedly different risk of morbidity and mortality [11].

## Conclusions

Important knowledge on the pathophysiology of septic shock has been built up over decades of experimental and clinical research. Translation of these scientific foundations into clinical practice has, however, been slow and erratic. For such a condition with a mortality risk of at least 30–40%, we should expect the rationale of consensus recommendations to be firmly grounded on pathophysiology. Our opinion is that some of the recent SSC’s hemodynamic recommendations move far away from this objective and might not constitute a valuable contribution to improve septic shock morbidity or mortality.

## Abbreviations

DAP: Diastolic arterial pressure
IV: Intravenous
P(cv-a)CO_2_: Carbon dioxide pressure difference between central vein blood and arterial blood
PCO_2_: Carbon dioxide partial pressure
ScvO_2_: Central venous oxygen saturation
SSC: Surviving Sepsis Campaign
